# The Associated Ion between the VDR Gene Polymorphisms and Susceptibility to Hepatocellular Carcinoma and the Clinicopathological Features in Subjects Infected with HBV

**DOI:** 10.1155/2013/953974

**Published:** 2013-03-23

**Authors:** Xing Yao, Huazong Zeng, Guolei Zhang, Weimin Zhou, Qiang Yan, Licheng Dai, Xiang Wang

**Affiliations:** ^1^Huzhou Central Hospital, Huzhou, Zhejiang 313000, China; ^2^School of Life Sciences and Technology, Tongji University, Shanghai 200092, China

## Abstract

*Aim*. To evaluate the possible association between the vitamin D receptor (VDR), single-nucleotide polymorphisms (SNPs), and hepatocellular carcinoma (HCC) in patients with chronic hepatitis B virus (HBV) infection. *Method*. 968 chronic HBV infection patients were enrolled, of which 436 patients were diagnosed HCC patients, and 532 were non-HCC patients. The clinicopathological characteristics of HCC were evaluated. The genotypes of VDR gene at FokI, BsmI, ApaI, and TaqI were determined. *Results*. The genotype frequencies of VDR FokI C>T polymorphism were significantly different between HCC and non-HCC groups. HCC patients had a higher prevalence of FokI TT genotype than non-HCC subjects. With FokI CC as reference, the TT carriage had a significantly higher risk for development of HCC after adjustments with age, sex, HBV infection time, **α**-fetoprotein, smoking status, and alcohol intake. In addition, we also found that the TT genotype carriage of FokI polymorphisms were associated with advanced tumor stage, presence of cirrhosis, and lymph node metastasis. The SNP at BsmI, ApaI, and TaqI did not show positive association with the risk and clinicopathological features of HCC. *Conclusion*. The FokI C>T polymorphisms may be used as a molecular marker to predict the risk and to evaluate the disease severity of HCC in those infected with HBV.

## 1. Introduction

Hepatocellular carcinoma (HCC) is one of the most common malignancies worldwide and the second leading cause of cancer-related death in China [1, 2]. The carcinogenesis of HCC is a multifactor, multistep, and complex process. It is known that multiple risk factors, including chronic hepatitis B virus (HBV) or hepatitis C virus (HCV) infection, cirrhosis, carcinogen exposure, and excessive alcohol consumption, contribute to hepatocarcinogenesis [3–5]. Epidemiological studies also showed that a variety of genetic factors mediate an individual's susceptibility to cancer [6–8]. The identification of genetic factors related to HCC susceptibility may help to elucidate the complex process of hepatocarcinogenesis and improve the scientific basis for preventative intervention.

The vitamin D receptor (VDR) is a member of the nuclear receptor superfamily of ligand-inducible transcription factors, which are involved in many physiological processes, including cell growth and differentiation, embryonic development, and metabolic homeostasis. The role of VDR in cancer has recently attracted much attention [9–11]. 

 Several single nucleotide polymorphisms (SNP) have been described in the VDR gene, and some SNPs are associated with tumor development. For instance, VDR polymorphisms have been related to the risk and prognosis of breast, prostate, skin, colon rectum, bladder and renal cell carcinoma, and malignant melanoma [12–16]. VDR polymorphisms have been investigated in the context of some chronic liver diseases, such as primary biliary cirrhosis and autoimmune hepatitis [17–19]. In a very recent published study, VDR genetic polymorphisms are significantly associated with the occurrence of HCC in Caucasian patients with alcoholic liver cirrhosis [20]. In China, hepatitis virus infection is a highly endemic factor for HCC [21–24]. However, the association between the VDR gene polymorphisms and the risk and pathological development in Chinese subjects with chronic hepatitis virus infection remains unknown. This case-control study, therefore, aimed to evaluate the role of VDR gene SNPs in the susceptibility and clinicopathological status of HCC in Chinese subjects with chronic HBV infection.

## 2. Methods

### 2.1. Subjects and Specimen Collection

This is a hospital-based case-control study. A total of 968 chronic HBV infection patients were enrolled at our hospital between Jan 2006 and Mar 2010. Of 968 patients, 436 patients were recruited as a HCC group, and 532 were enrolled as non-HCC group according to the presence or absence of HCC. We also enrolled 132 patients with determined HCC, but without HBV infection as control. The patients were diagnosed with HCC based on the characteristic criteria of the national guidelines for HCC, such as liver injury diagnosed by either histology or cytology irrespective of *α*-fetoprotein (AFP) titer where imaging data showed any one of following three items: (1) one or more liver masses ≥2 cm in diameter via both computed tomography and magnetic resonance imaging; (2) imaging data with early enhancement and a high level of AFP ≥400 ng/mL; (3) imaging data with early arterial phase contrast enhancement plus early venous phase contrast washout regardless of AFP level. Liver cirrhosis was determined by histology, imaging, or clinical indications, such as esophageal varices or ascites. Age, gender, smoke status, and alcohol use were recorded. Relevant medical information including stage of HCC, liver cirrhosis history, lymph node metastasis, portal invasion, AFP, aspartate aminotransferase (AST), and alanine aminotransferase (ALT) was also collected from patients by medical chart review. Heavy alcohol intake was defined as ethanol intake ≥80 g/day for >10 years. Patients with other cancers and chronic diseases, especially kidney, bone metabolism diseases were strictly excluded. This study was approved by the Institutional Review Board of our Hospital, and written informed consent was obtained from all study subjects.

### 2.2. DNA Extraction and Genotyping

Genomic DNA was extracted using QIAamp DNA blood mini kits (Qiagen, Valencia, CA) following the manufacturer's instructions. For the detection of the VDR polymorphisms, the polymerase chain reaction (PCR) technique was applied and followed by restriction fragment length polymorphism assays. The primer sequences were shown in Table 1. The cycling conditions for all the VDR polymorphisms were set as 40 cycles at 95°C for 30 s, 61°C for 30 s, and 72°C for 1 min. In a total volume of 20 *μ*L, amplified DNA (10 *μ*L) was digested overnight with 2 U of restriction endonucleases using the buffers and temperatures recommended by the manufacturers. All the PCR products were sized by electrophoresis on a 2% agarose gel stained with ethidium bromide.

### 2.3. Statistical Analysis

The distributions of demographic characteristics and genotype frequencies between the cases and controls and the clinicopathological features in different genotypes were analyzed by Fisher's exact test, since the small sample size was present in some categories of variables. Hardy-Weinberg equilibrium was assessed using *χ* ^2^ test. The odds ratios (ORs) with their 95% confidence intervals (CIs) of the association between genotype frequencies and HCC were estimated by multiple logistic regression models, after controlling for other covariates, including age, gender, and genotypes for each estimated variable. A *P* value of less than 0.05 was considered significant. The data were analyzed on SAS statistical software (Version 9.1, 2005; SAS Institute Inc., Cary, NC).

## 3. Results

The characteristics of the study population are presented in Table 2. There were no significant differences of age, gender, HBV infection time, smoker status, and family cancer history between HCC and non-HCC. However, HCC group had a significantly higher rate of heavy alcohol intake compared with controls (*P* = 0.034). We observed a significant difference in BMI between HCC and non-HCC subjects. The mean serum AFP levels were significantly higher in HCC subjects than non-HCC subjects (all *P* < 0.001). HCC patients had a significantly higher rate of liver cirrhosis compared with non-HCC patients (*P* < 0.001). 

The genotype frequencies of VDR gene in HCC and non-HCC subjects are presented in Table 3. The genotype frequencies of all SNPs in control patients were in Hardy-Weinberg equilibrium (all *P* > 0.05). The genotype frequencies of VDR FokI C>T polymorphism were significantly different between HCC and non-HCC groups. HCC patients had a higher prevalence of FokI TT genotype than non-HCC subjects (30.05% versus 19.17%, *P* < 0.001). For allele comparison, HCC subjects had higher T allele frequency than controls (52.75% versus 41.82%, *P* < 0.001). To identify the independent risk factor for the development of HCC, we performed the multivariate regression analyses. With FokI CC as reference, our data showed that the TT carriage had a significantly higher risk for development of HCC after adjustments with age, gender, HBV infection time, smoker status, family cancer history, alcohol intake, BMI, and serum AFP level (OR = 2.269, *P* = 0.006). With C allele as reference, the OR of T allele carriage for HCC was 1.553 (*P* < 0.001). For SNPs of BsmI, ApaI, and TaqI, their genotype and allele frequencies did not significantly differ between HCC and non-HCC group (all *P* > 0.05). Multivariate regression analyses showed no association between the above-mentioned SNPs of VDR gene and susceptibility of HCC in this study.

Table 4 showed the genotype frequencies of VDR gene in HCC patients with chronic HBV infection and HCC patient without HBV infection. We found that the VDR genotype frequencies were similar between HCC with HBV infection and HCC without HBV infection. None of the VDR SNPs showed significant difference between HCC with HBV infection and HCC without HBV infection (All *P* > 0.05). 

We further analyzed the association between VDR polymorphisms and the clinicopathological features in HCC subjects (Table 5). We found that only the SNPs at FokI locus were significantly different when all HCC patients were stratified by tumor stage, presence of liver cirrhosis history, and lymph node metastasis (all *P* < 0.05), but not tumor size and portal invasion. We further performed the multivariate regression analyses to explore the role of SNPs at FokI locus in determining the clinicopathological features in HCC subjects. Taking the CC genotype as reference, we found that the TT genotype carriage of FokI polymorphisms was associated with advanced tumor stage (OR for III + IV stage = 2.335, *P* = 0.001), presence of cirrhosis (OR for presence = 2.714, *P* < 0.001), and lymph node metastasis (OR for presence of lymph node metastasis = 2.122, *P* = 0.004). No association between the FokI polymorphisms, tumor size, and portal invasion was observed (both adjusted *P* > 0.05). The SNP at other loci including BsmI, ApaI, and TaqI did not show positive association with the the clinicopathological features of HCC (data not shown). 

**Figure 1:**
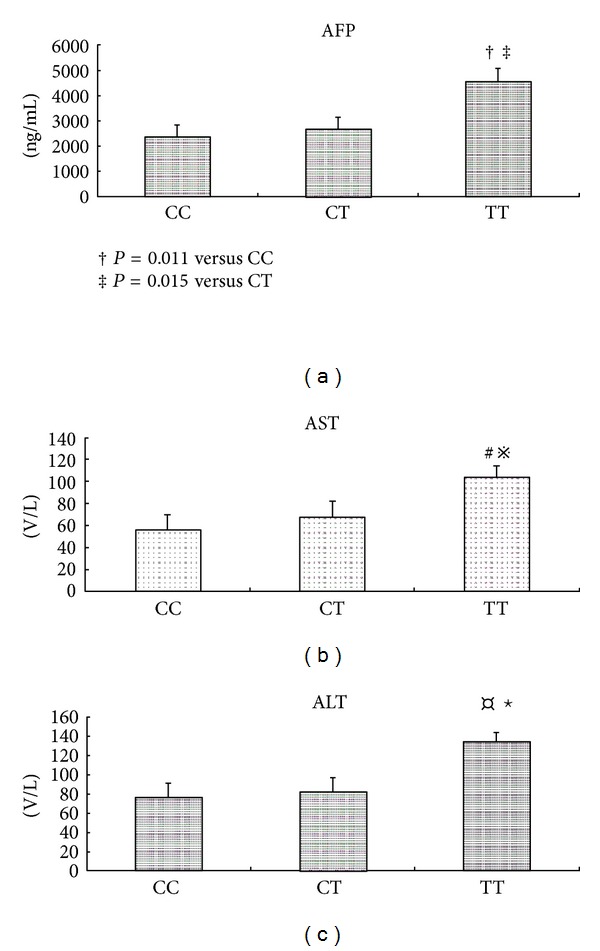
Serum AFP, AST, and ALT levels among different VDR FokI C>T genotype carriers in HCC patients*. *

AFP is the common clinical pathological markers of HCC. We compared the serum AFP levels among different VDR genotype carriers in HCC patients. We found HCC patients with FokI TT genotype had a much higher AFP level than CT and CC carriers (Figure 1(a), *P* = 0.011 versus CC; *P* = 0.015 versus CT, resp.). The AFP levels were similar among BsmI, ApaI, and TaqI genotype carriers (data not shown). We also compared the liver function marker, namely, serum ALT and AST level according to the VDR genotypes. We found that only the FokI TT carriers had higher serum AST and ALT levels compared with TC and CC (all *P* < 0.05, Figures 1(b) and 1(c)) in HBV infection patient with HCC, but not in the HBV infection patients without HCC (data not shown).

## 4. Discussion

In the study, we investigated the possible association between the VDR gene polymorphisms and HCC in a Chinese population with HBV infection. We found that the FokI C>T polymorphisms was significantly associated with the susceptibility and clinical features of HCC, including advanced tumor stage, presence of liver cirrhosis history, and lymph node metastasis. Since the association between the VDR genetic polymorphism and HCC had not been previously reported, our data, for the first time, provide new information in this aspect. The results of this study suggest the FokI C>T polymorphisms may be used as a molecular marker to predict the risk and to evaluate the disease severity of HCC in those infected with HBV. 

Hepatitis virus infection is associated with the increase of oxidative stress in liver cells and results in DNA changes and instability, thus increasing the risk of developing cirrhosis and/or HCC [21, 25]. Some previous studies provided evidence that genetic polymorphisms of certain genes may predict the HCC occurrence in hepatitis virus infection [26–29]. 

The VDR is an intracellular hormone receptor that specifically binds the biologically active form of vitamin D, 1,25-dihydroxyvitamin D or calcitriol and interacts with specific nucleotide sequences (response elements) of target genes to produce a variety of biologic effects [30]. The VDR gene is located on chromosome 12q12–q14, and several single-nucleotide polymorphisms have been identified that may influence cancer risk [31].

The FokI restriction fragment length polymorphism, located in the coding region of the VDR gene, results in the production of a VDR protein that is three amino acids longer and functionally less effective [32]. Arai et al. demonstrated that compared with the FokI T/T genotype, FokI C/C had 1.7-fold greater function of vitamin D-dependent transcriptional activation of a reporter construct under the control of a vitamin D response element in transfected HeLa cells [33]. It has been hypothesized that a less active VDR could be associated with either an increased susceptibility to cancer risk or to a more aggressive disease [31]. 

Vogel et al. detected a significant association between the F allele of the FokI C>T (F/f) polymorphism and autoimmune hepatitis patients, indicating a genetic link of VDR polymorphisms to autoimmune liver diseases such as primary biliary cirrhosis in German patients [19]. In Chinese population, Fan et al. reported a significant difference in FokI polymorphism between autoimmune hepatitis patients and controls, and a significant association in BsmI polymorphisms between primary biliary cirrhosis patients and controls [34]. In Italian population who underwent liver transplantation with the etiologies of liver disease as hepatitis C, hepatitis B, and alcoholic liver disease, carriage of the GG genotype of BsmI and the TT genotype of TaqI was significantly associated with occurrence of HCC. The authors concluded that VDR genetic polymorphisms are significantly associated with the occurrence of HCC in patients with liver cirrhosis, and this relationship is more specific for patients with an alcoholic etiology [20]. Our study showed that only the FokI C>T polymorphisms were associated with the HCC susceptibility in Chinese patient with chronic HBV infection; the other SNPs, including BsmI, were not related with the development of HCC. The HCC patients had a significantly higher cirrhosis rate than non-HCC patients (*P* < 0001), suggesting that the VDR polymorphisms at FokI locus may relate to HCC risk by enhancing the occurrence of liver cirrhosis. Although many factors may be account for the discrepancies among these studies, the ethnic difference should be predominately considered since Fan et al. found the distribution of FokI, BsmI, ApaI, and TaqI gene types significantly differed between Chinese healthy controls and Caucasian healthy controls [34]. 

The prognostic role of FokI C>T (F/f) polymorphism in cancer patients has been reported. Hama et al. reported the VDR FokI TT genotype was associated with a poor progression-free survival rate in patients with head and neck squamous cell carcinoma. In contrast, the other polymorphisms (BsmI, ApaI, and TaqI) showed no significant associations with progression-free survival [35]. In this study, we did not perform prognostic analyses; however, we found that the TT genotype carriage of TaqI polymorphisms was associated with advanced tumor stage, worse tumor differentiation, presence of cirrhosis, and lymph node metastasis. In addition, the TT carriers had higher serum AFP level than CC and CT carriers. The SNP at other loci including BsmI, ApaI, and TaqI did not show positive association with the clinicopathological features of HCC. Since tumor stage, tumor differentiation, presence of cirrhosis, and lymph node metastasis are conventional factors associated with HCC prognosis, our data imply that the TT carriers had poorer prognosis than CC and CT carriers. 

Some limitation in this study should be addressed. Firstly, this is a hospital-based case-control study, thus the enrollment bias was inevitable. Secondly, no information was available regarding vitamin D intake (dietary or supplemental) and circulating vitamin D levels of patients. Thirdly, we only enrolled subjects with HBV infection, and all the subjects were Chinese. As HCV infection is another major cause for HCC in China, future study with larger sample size including HCV subjects is warranted.

## Figures and Tables

**Table 1:** The primer sequences of VDR gene polymorphism at 4 loci.

SNP	Primer	Base change	*T*
FokI	5′AGCTGGCCCTGGCACTGACTCTGCTCT3′ (F)	C>T	61°C
	5′ATGGAAACACCTTGCTTCTTCTCCCTC3′ (R)		
BsmI	5′CAACCAAGACTACAAGTACCGCGTCAGTGA3′ (F)	G>A	57°C
	5′AACCAGCGGGAAGAGGTCAAGGG3′ (R)		
ApaI	5′CAGAGCATGGACAGGGAGCAA3′ (F)	G>T	60°C
	5′GCAACTCCTCATGGCTGAGGTCTC3′ (R)		
TaqI	5′CAGAGCATGGACAGGGAGCAA3′ (F)	T>C	60°C
	5′GCAACTCCTCATGGCTGAGGTCTC3′ (R)		

**Table 2:** The characteristics of the study population.

	HCC		Non-HCC		*P*
Age (years)	52.45 ± 4.6		51.95 ± 2.8		NS
Sex (male, %)	75.40%		74.84%		NS
HBV infection time (month)	15.4 ± 3.4		13.8 ± 4.6		0.053
BMI (kg/m^2^)	20.4 ± 2.4		22.1 ± 1.9		0.03
Serum AFP (ng/mL)	3546 ± 224		—		

	*n*	%	*n*	%	

Heavy alcohol intake, *n* (%)					
Yes	152	34.86%	109	20.49%	0.034
No	284	65.14%	423	79.51%	
Smoking status, *n* (%)			532		
Smokers	122	27.98%	98	22.69%	
Nonsmokers	314	72.02%	334	77.31%	NS
Family cancer history					
Yes	72	16.51%	90	16.92%	NS
No	364	83.49%	442	83.08%	
Liver cirrhosis *n* (%)					
Yes	213	48.85%	195	36.65%	<0.001
No	223	51.15%	337	63.35%	

**Table 3:** The genotype frequencies of VDR gene in HCC and non-HCC among patients with chronic HBV infection.

	HCC		Non-HCC					
					Adjusted OR	95% CI		Adjusted *P* value
	*N*	%	*N*	%				
BsmI								
GG	112	25.69%	142	26.69%	1			
GA	217	49.77%	259	48.68%	1.062	0.782	1.443	0.699
AA	107	24.54%	131	24.62%	1.036	0.726	1.478	0.847
G	441	50.57%	543	51.03%	1			
A	431	49.43%	521	48.97%	1.019	0.852	1.218	0.84
FokI								
CC	107	24.54%	189	35.53%	1			
CT	198	45.41%	241	45.30%	1.451	1.072	1.964	0.016
TT	131	30.05%	102	19.17%	2.269	1.597	3.223	0.006
C	412	47.25%	619	58.18%	1			
T	460	52.75%	445	41.82%	1.553	1.297	1.86	<0.001
ApaI								
GG	108	24.77%	143	26.88%	1			
GT	216	49.54%	275	51.69%	1.04	0.765	1.414	0.802
TT	112	25.69%	114	21.43%	1.301	0.907	1.867	0.165
G	432	49.54%	561	52.73%	1			
T	440	50.46%	503	47.27%	1.136	0.95	1.359	0.163
TaqI								
TT	115	26.38%	137	25.75%	1			
TC	212	48.62%	252	47.37%	1	0.741	1.363	0.993
CC	109	25.00%	143	26.88%	0.915	0.644	1.279	0.592
T	442	50.69%	526	49.44%	1			
C	430	49.31%	538	50.56%	0.952	0.823	1.145	0.581

**Table 4:** The genotype frequencies of VDR gene in HCC patients with chronic HBV infection and HCC patient without HBV infection.

	HCC with HBV		HCC without HBV		Adjusted OR	95% CI		Adjusted *P* value
BsmI								
GG	112	25.69%	37	28.24%	1.000			
GA	217	49.77%	65	49.62%	1.103	0.694	1.753	0.679
AA	107	24.54%	29	22.14%	1.219	0.701	2.120	0.483
G	441	50.57%	139	53.05%	1.000			
A	431	49.43%	123	46.95%	1.104	0.838	1.456	0.481
FokI								
CC	107	24.54%	31	23.66%	1.000			
CT	198	45.41%	62	47.33%	0.925	0.566	1.512	0.756
TT	131	30.05%	38	29.01%	0.999	0.583	1.712	0.745
C	412	47.25%	124	47.33%	1.000			
T	460	52.75%	138	52.67%	1.003	0.761	1.323	0.982
ApaI								
GG	108	24.77%	22	16.79%	1.000			
GT	216	49.54%	75	57.25%	0.587	0.346	0.995	0.052
TT	112	25.69%	34	25.95%	0.671	0.369	1.220	0.571
G	432	49.54%	119	45.42%	1.000			
T	440	50.46%	143	54.58%	0.848	0.642	1.118	0.242
TaqI								
TT	115	26.38%	32	24.43%	1.000			
TC	212	48.62%	69	52.67%	0.855	0.531	1.377	0.519
CC	109	25.00%	30	22.90%	1.011	0.576	1.775	0.970
T	442	50.69%	133	50.76%	1.000			
C	430	49.31%	129	49.24%	1.003	0.761	1.322	0.983

**Table 5:** Association between VDR polymorphisms and the clinicopathological features in HCC subjects.

FokI C>T		Tumor size			Adjusted OR	95% CI		Adjusted *P* value

	<30 mm	%	>30 mm	%				
CC	55	25.00%	57	26.39%	1			
CT	108	49.09%	109	50.46%	1.027	0.651	1.62	0.543
TT	57	25.91%	50	23.15%	1.181	0.695	2.008	0.068

		Cancer stage						
	III + IV		I + II					

CC	43	18.53%	64	29.91%	1			
CT	109	46.98%	99	46.26%	1.639	1.021	2.629	0.054
TT	70	34.48%	51	23.83%	2.335	1.385	3.936	0.001

		Liver cirrhosis history						
	Presence		Absence					

CC	40	18.78%	67	30.04%	1			
CT	92	43.19%	106	47.53%	1.454	0.899	2.352	0.127
TT	81	38.03%	50	22.42%	2.714	1.602	4.596	<0.001

		Lymph node metastasis						
	Presence		Absence					

CC	43	18.45%	64	29.77%	1			
CT	96	41.20%	102	47.44%	1.401	0.87	2.256	0.038
TT	77	33.05%	54	25.12%	2.122	1.262	3.57	0.004

		Portal invasion						
	Presence		Absence					

CC	53	25.48%	62	27.19%	1			
CT	110	52.88%	102	44.74%	1.260	0.843	1.991	0.326
TT	45	21.63%	64	28.07%	0.820	0.482	1.411	0.470

		Heavy alcohol intake, *n* (%)						
	Yes		No					

CC	43	28.29%	67	27.02%	1.000			
CT	75	49.34%	126	50.81%	0.927	0.575	1.496	0.757
TT	34	22.37%	55	22.18%	0.963	0.543	1.710	0.898

## References

[1] Małkowski P, Pacholczyk M, Łagiewska B (2006). Hepatocellular carcinoma—epidemiology and treatment. *Przeglad Epidemiologiczny*.

[2] van den Bosch MA, Defreyne L (2012). Hepatocellular carcinoma. *The Lancet*.

[3] Farazi PA, DePinho RA (2006). The genetic and environmental basis of hepatocellular carcinoma. *Discovery Medicine*.

[4] Masuzaki R, Yoshida H, Tateishi R, Shiina S, Omata M (2008). Hepatocellular carcinoma in viral hepatitis: improving standard therapy. *Best Practice and Research: Clinical Gastroenterology*.

[5] Singal AK (2008). Silent cirrhosis in hepatitis B virus related hepatocellular carcinoma. *Hepato-Gastroenterology*.

[6] Wang PR, Xu M, Toffanin S, Li Y, Llovet JM, Russell DW (2012). Induction of hepatocellular carcinoma by in vivo gene targeting. *Proceedings of the National Academy of Sciences of the United States of America*.

[7] Ma NF, Lau SH, Hu L, Dong SS, Guan XY (2011). Hepatitis B virus X gene in the development of hepatocellular carcinoma. *Hong Kong Medical Journal*.

[8] Cillo C, Schiavo G, Cantile M (2011). The HOX gene network in hepatocellular carcinoma. *International Journal of Cancer*.

[9] Yee YK, Chintalacharuvu SR, Lu J, Nagpal S (2005). Vitamin D receptor modulators for inflammation and cancer. *Mini-Reviews in Medicinal Chemistry*.

[10] Guy M, Lowe LC, Bretherton-Watt D (2004). Vitamin D receptor gene polymorphisms and breast cancer risk. *Clinical Cancer Research*.

[11] Slattery ML (2007). Vitamin D receptor gene (VDR) associations with cancer. *Nutrition Reviews*.

[12] Denzer N, Vogt T, Reichrath J (2011). Vitamin D receptor (VDR) polymorphisms and skin cancer: a systematic review. *Dermato-Endocrinology*.

[13] Buyru N, Tezol A, Yosunkaya-Fenerci E, Dalay N (2003). Vitamin D receptor gene polymorphisms in breast cancer. *Experimental and Molecular Medicine*.

[14] Blazer DG, Umbach DM, Bostick RM, Taylor JA (2000). Vitamin D receptor polymorphisms and prostate cancer. *Molecular Carcinogenesis*.

[15] Murtaugh MA, Sweeney C, Ma KN (2006). Vitamin D receptor gene polymorphisms, dietary promotion of insulin resistance, and colon and rectal cancer. *Nutrition and Cancer*.

[16] Zhou W, Heist RS, Liu G (2006). Polymorphisms of vitamin D receptor and survival in early-stage non-small cell lung cancer patients. *Cancer Epidemiology Biomarkers and Prevention*.

[17] Kempinska-Podhorecka A, Wunsch E, Jarowicz T (2012). Vitamin D receptor polymorphisms predispose to primary biliary cirrhosis and severity of the disease in polish population. *Gastroenterology Research and Practice*.

[18] Tanaka A, Nezu S, Uegaki S (2009). Vitamin D receptor polymorphisms are associated with increased susceptibility to primary biliary cirrhosis in Japanese and Italian populations. *Journal of Hepatology*.

[19] Vogel A, Strassburg CP, Manns MP (2002). Genetic association of vitamin D receptor polymorphisms with primary biliary cirrhosis and autoimmune hepatitis. *Hepatology*.

[20] Falleti E, Bitetto D, Fabris C (2010). Vitamin D receptor gene polymorphisms and hepatocellular carcinoma in alcoholic cirrhosis. *World Journal of Gastroenterology*.

[21] Tan YJ (2011). Hepatitis B virus infection and the risk of hepatocellular carcinoma. *World Journal of Gastroenterology*.

[22] Hou J, Liu Z, Gu F (2005). Epidemiology and prevention of hepatitis B virus infection. *International Journal of Medical Sciences*.

[23] Moriyama M, Mikuni M, Longren W (2003). Epidemiology of SEN virus infection among patients with hepatitis B and C in China. *Hepatology Research*.

[24] Merican I, Guan R, Amarapuka D (2000). Chronic hepatitis B virus infection in Asian countries. *Journal of Gastroenterology and Hepatology*.

[25] Jin Y, Abe K, Sato Y, Aita K, Irie H, Shiga J (2002). Hepatitis B and C virus infection and p53 mutations in human hepatocellular carcinoma in Harbin, Heilongjian Province, China. *Hepatology Research*.

[26] Migita K, Maeda Y, Abiru S (2007). Polymorphisms of interleukin-1*β* in Japanese patients with hepatitis B virus infection. *Journal of Hepatology*.

[27] Al-Qahtani A, Al-Ahdal M, Abdo A (2012). Toll-like receptor 3 polymorphism and its association with hepatitis B virus infection in Saudi Arabian patients. *Journal of Medical Virology*.

[28] Kim JH, Yu SJ, Park BL (2011). TGFBR3 polymorphisms and its haplotypes associated with chronic hepatitis B virus infection and age of hepatocellular carcinoma occurrence. *Digestive Diseases*.

[29] Chan KY, Wong CM, Kwan JS (2011). Genome-wide association study of hepatocellular carcinoma in Southern Chinese patients with chronic hepatitis B virus infection. *PLoS One*.

[30] Gross C, Krishnan AV, Malloy PJ, Eccleshall TR, Zhao XY, Feldman D (1998). The vitamin D receptor gene start codon polymorphism: a functional analysis of FokI variants. *Journal of Bone and Mineral Research*.

[31] Whitfield GK, Remus LS, Jurutka PW (2001). Functionally relevant polymorphisms in the human nuclear vitamin D receptor gene. *Molecular and Cellular Endocrinology*.

[32] Uitterlinden AG, Fang Y, van Meurs JBJ, Pols HAP, van Leeuwen JPTM (2004). Genetics and biology of vitamin D receptor polymorphisms. *Gene*.

[33] Arai H, Miyamoto KI, Taketani Y (1997). A vitamin D receptor gene polymorphism in the translation initiation codon: effect on protein activity and relation to bone mineral density in Japanese women. *Journal of Bone and Mineral Research*.

[34] Fan L, Tu X, Zhu Y (2005). Genetic association of vitamin D receptor polymorphisms with autoimmune hepatitis and primary biliary cirrhosis in the Chinese. *Journal of Gastroenterology and Hepatology*.

[35] Hama T, Norizoe C, Suga H (2011). Prognostic significance of vitamin D receptor polymorphisms in head and neck squamous cell carcinoma. *PLoS One*.

